# The real role of pseudokinase: linking diabetes to cancers

**DOI:** 10.1002/cam4.706

**Published:** 2016-03-31

**Authors:** Ke Li, Zhuowei Hu

**Affiliations:** ^1^ Immunology and Cancer Pharmacology GroupState Key Laboratory of Bioactive Substance and Function of Natural MedicinesInstitute of Materia Medica, Peking Union Medical CollegeBeijing100050 China; ^2^ Institute of Medicinal BiotechnologyChinese Academy of Medical SciencesPeking Union Medical CollegeBeijing100050China

**Keywords:** Autophagy, protein–protein interaction, SQSTM1, TRIB3, UPS, *α*‐helix peptide

## Abstract

A recent paper in Nature Communications shows that pseudokinase TRIB3 has a critical role in the development of diabetes‐related cancers via interacting with SQSTM1, a selective autophagy receptor. Interrupting the TRIB3‐SQSTM1 interaction using an *α*‐helix peptide shows a significant antitumor effect both in normal and diabetic mice. This work provides a potential strategy against cancers in patients with diabetes.

Type 2 diabetes (T2D) and cancer are two chronic diseases with enormous impact on health worldwide. Epidemiologic and clinical studies suggest that people with diabetes are at a higher risk for multiple primary cancers. Indeed, medications used to treat diabetes are associated with either an increased or decreased risk of cancer depending on their enhancing or reducing circulating levels of blood glucose and insulin 1, 2. T2D and cancer share a number of metabolic risk factors, including high insulin/IGF, hyperglycemia, glucose deprivation, hypoxia and inflammation, but the underlined mechanism remains unclear. In a recent report 3, Hu and his colleagues have verified that TRIB3, a stress‐induced protein, links metabolic risk factors to cancer development and progression via interacting with SQSTM1, a selective autophagy receptor. Interrupting the TRIB3‐SQSTM1 interaction using an *α*‐helix peptide shows a significant antitumor effect both in normal and diabetic mice (Fig. 1). The authors’ work provides a potential strategy against cancers in patients with diabetes.

**Figure 1:**
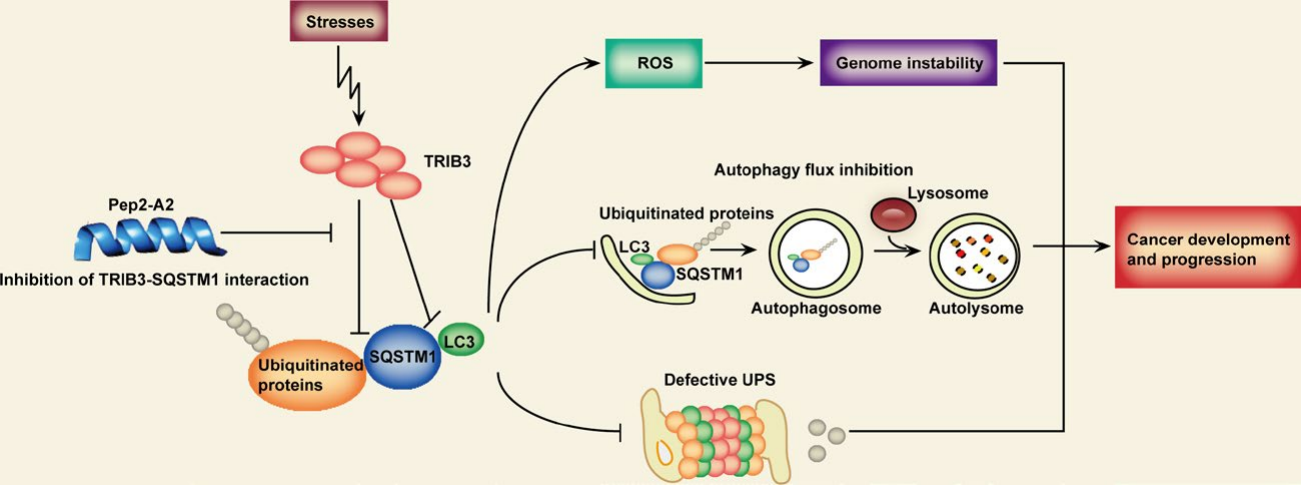
The role of TRIB3 in metabolic stresses induced cancer development and progression. Metabolic stresses increase TRIB3 expression to promote the interaction of TRIB3 and SQSTM1. The TRIB3‐SQSTM1 interaction interferes with the binding of LC3 and ubiquitinated proteins to SQSTM1, which induces the autophagic flux inhibition, subsequently defective ubiquitin proteasome system (UPS) and the accumulation of ROS. ROS accumulation and dysfunction of two degradation systems result in a genome instability and a deposition of cancer‐promoting factors. TRIB3‐binding *α*‐helical peptide (Pep2‐A2) can interfere with the TRIB3‐SQSTM1 interaction and produce a potent antitumor effect, suggesting that targeting TRIB3/SQSTM1 interaction is a therapeutic strategy against diabetes‐related cancers.

TRIB3 is an evolutionarily conserved member of pseudokinases, which plays important roles in proliferation, metabolism and oncogenic transformation 4. In addition, modulation of PI3K/AKT signaling pathway highlights TRIB3 as a novel and rather unusual therapeutic target. Moreover, metabolic risk factors exert oncogenic actions via the activation of the PI3K/AKT/mTOR and MAPK/ERK pathways. Hence, these authors find that there is a positive correlation between TRIB3 and activation of insulin signaling in tumor tissues. Several metabolic risk factors, including insulin and IGF‐1‐enhanced TRIB3 expression in a diversity of human cancer cells. TRIB3 depletion not only protects against the tumor‐promoting actions of insulin/IGF in cancer cells, but also suppresses tumor initiation, growth and metastasis in diabetic mice. Their results suggest that TRIB3 plays a crucial role in tumorigenesis and tumor progression in mice, particularly in diabetic mice.

The PI3K/AKT/mTOR pathway contributes to cancer progression through the modulation of autophagy. TRIB3 can suppress the phosphorylation of AKT, a negative modulator of autophagy. However, these researchers found that TRIB3 overexpression inhibits LC3‐I/‐II conversion and increases the level of both soluble and insoluble SQSTM1 in cancer cells. In contrast, silencing TRIB3 decreases the basal and IGF‐1‐induced accumulation of SQSTM1 in cancer cells. Because the SQSTM1 protein level is one of critical indicators of autophagic flux, TRIB3 depletion inducing SQSTM1 clearance leads to an activation of the autophagy‐dependent degradation pathway. They further find that silencing TRIB3 not only decreases the basal levels of critical tumor‐promoting factors, but also protects them from the IGF‐1 induction. Hence, their results further indicate that TRIB3 acts as a proto‐oncogene sustaining the cancer cells a feature of cancer‐initiating phenotype.

TRIB3 is uniquely defined by a pseudokinase domain containing an *α* C‐helix, a MEK‐binding site and a distinct C‐terminal peptide motif which interacts directly with different kinds of proteins. Their previous study revealed that TRIB3 plays a critical role in TGF*β*‐mediated cancer metastasis by interacting with the signaling molecule SMAD3 5. In this recent report, Hu and colleagues find that TRIB3 induces SQSTM1 accumulation and dysfunction by interacting with the LIR motif and UBA domain of SQSTM1, leading to the dysfunction of SQSTM1‐mediated autophagy and this reduces degradation of ubiquitinated proteins. Moreover, the SQSTM1 accumulation and dysfunction of autophagy caused by TRIB3 inhibit Ubiquitin Proteasome System (UPS)‐dependent substrate clearance (Fig. 1). Therefore, they conclude that the TRIB3/SQSTM1 interaction mediates the IGF‐induced the SQSTM1 accumulation, which compromises UPS as well as autophagic degradation in cancer cells.

Protein–protein interactions (PPIs) are indispensable to majority of biological functions and PPIs may act as a novel and highly promising class of drug targets. However, there are a number of challenges and concerns regarding PPIs targets, some of which include large PPIs interface areas, a lack of deep pockets, and the presence of noncontiguous‐binding sites 6. Hence, a small molecule could hardly dock into the interface surfaces of many PPIs. However, structural studies permit the identification of peptide fragments and amino acid residues which are critical for PPIs. Moreover, mimicking the structure of binding peptides is one of the approaches widely used to design novel PPIs modulators. Several PPI modulators that inhibit the MDM2/p53, XIAP/caspase‐9 or BCL2/beclin1 interaction are undergoing clinical trials for different cancers.

In this study, they screen a TRIB3‐binding *α*‐helical peptide (A2) from the SQSTM1's UBA domain which is involved in the TRIB3/SQSTM1 interaction using the surface plasmon resonance method. They further find that peptide A2 specifically inhibits the interaction of TRIB3/SQSTM1 in vitro. However, the difficulty of cellular uptake has hindered A2 to exert its interrupting the TRIB3/SQSTM1 interaction in cancer cells. In their previous study, they identified a cell penetrating peptide, Pep2, which could deliver large molecules, including peptides and nucleic acids into cancer cells through the cell membrane 7.In this report, a fused peptide Pep2‐A2 has been designed by linking a cell‐penetrating peptide to A2, using a glycine–glycine linker. They find that Pep2‐A2 displays a significant inhibition of SQSTM1/TRIB3 interaction in tumor cells, rescuing the TRIB3‐reduced association of SQSTM1 with LC3 and ubiquitinated proteins. Moreover, treatment of cancer cells with Pep2‐A2 activates autophagic flux and UPS, and decreases the expression of several tumor‐promoting factors. Interestingly, the Pep2‐A2 treatment obviously attenuates tumor growth and metastasis in vivo, especially in diabetic mice. Hu et al. thought that the therapeutic efficacy of Pep2‐A2 against tumors may not only result from the effects of the peptide on SQSTM1/TRIB3 interaction, but also the following effects on several other protein–protein interactions, activation of autophagy and UPS. Therefore, more studies should be carried out to reveal the exact mechanisms of Pep2‐A2 for its antitumor effects.

Overall, their studies prove that stress protein TRIB3 mediates metabolic risk factors‐induced tumor development and progression. This work provides the proof‐of‐concepts for targeting the TRIB3/SQSTM1 interaction as a therapeutic strategy against cancers, especially in T2D patients with cancers.

## Conflict of Interest

The authors made no disclosures.
